# Retrospective evaluation of the epidemiological importance of *Triatoma infestans* and *Panstrongylus megistus* in the transmission of *Trypansoma cruzi* in a region of southeastern Brazil

**DOI:** 10.1590/S1678-9946202567079

**Published:** 2025-11-14

**Authors:** Nilvanei Aparecido da Silva Neves, Rita de Cássia Moreira de Souza, David Eladio Gorla, Lileia Gonçalves Diotaiuti

**Affiliations:** 1 Instituto René Rachou, Grupo de Pesquisa em Triatomíneos, Belo Horizonte, Minas Gerais, Brazil; 2 Universidad Nacional de Córdoba, Instituto de Diversidad y Ecología Animal, Grupo de Ecología y Control de Vectores, Córdoba, Argentina

**Keywords:** Chagas disease, Prevalence, Survey, Vector control

## Abstract

*Panstrongylus megistus* is the most important autochthonous vector of *Trypanosom cruzi*, the etiological agent of Chagas disease, in the midwest region of the Minas Gerais State, Brazil. This study investigates the vectorial roles of *Triatoma infestans* and *P. megistus* in Chagas disease in this geographical area during the late 1970s. A retrospective analysis of entomological and serological surveys from 1975–1983 was conducted, comparing the presence of *T. infestans* and *P. megistus* with the seroprevalence of *T. cruzi* infection in the human population within the Divinopolis Regional Health Superintendency. *Panstrongylus megistus* was recorded in all surveyed municipalities (52/52), whereas *T. infestans* co-occurrence with *P. megistus* was recorded in only 19.2% (10/52) of them. In the 41 municipalities where only *P. megistus* was found and relevant data were available, the mean seroprevalence of human *T. cruzi* infection was 17.8% ranging from 1.0% to 41.9%. In the municipalities where *T. infestans* occurred, the mean seroprevalence was higher, at 25.8%, ranging from 9.8% to 40.8%. Among the municipalities where only *P. megistus* was present, 19.5% had a low, 29.3% an intermediate, and 51.2% a high seroprevalence of human *T. cruzi* infection. In the ten municipalities where both *T. infestans* and *P. megitus* were found, 80% showed high seroprevalence, whereas only one municipality each showed low or intermediate levels. The findings highlight the significant role of *P. megistus* in *T. cruzi* transmission, even in the absence of *T. infestans*. The wide distribution of *P. megistus* increased the risk of transmission. *P. megistus* was the main household vector in the region in the 1970s. Nowadays, continuous surveillance remains essential for monitoring triatomine infestations and evaluating the current risk of *T. cruzi* transmission.

## INTRODUCTION

Triatominae (Hemiptera: Reduviidae) are insect vectors of the protozoa *Trypanosoma cruzi* (Chagas, 1909)^1^, the etiological agent of Chagas disease, an important illness that affects over seven million people across the Americas^2^. Currently, 158 triatomines species are recognized^3^. Vectorial transmission occurs when the insects feed on mammals and deposit infected feces on the skin, enabling the parasite to enter through breaks in the skin, eyes, or mouth^4^. In sylvatic environments, oral transmission is responsible for high infection rates among non-human vertebrates, particularly marsupials, rodents, and primates^5^, and has recently gained relevance in human transmission through the ingestion of contaminated food^6^.

In humans, Chagas disease gained greater epidemiological relevance following the domiciliation of triatomines, which invade and colonize human dwellings, forming large colonies and feeding on humans and their domestic animals^4,5^. This colonization often extends to the peridomestic environment, involving animals such as chickens, pigs, and other domestic or wild animals from these areas, thereby maintaining the transmission cycle near humans^4^. Consequently, vector control efforts target the epidemiological unit of the domicile, encompassing both the intra- and peridomestic areas^7^.

The ability to adapt to disturbed sylvatic and/or novel anthropogenic environments varies among triatomine species and is a major determinant of their importance as vectors. Triatomines of primary epidemiological relevance for *T. cruzi* transmission to humans are anthropophilic and form large indoor colonies. Those of secondary importance are mainly peridomestic, typically forming small indoor colonies. Other species are "invasive", which temporarily enter houses and intermittently transmit the parasite without establishing colonies^8^. Adaptation to anthropogenic habitats requires physiological adjustments that enable triatomines to feed off the blood of vertebrate hosts, different from those in their natural sylvatic environments^9^. In Brazil, triatomines species of primary importance include *Triatoma brasiliensis* (Neiva, 1911) in the semiarid Caatinga region of the Northeast and *Panstrongylus megistus* (Burmeister, 1835) in the Cerrado biome. However, extremely high prevalences of *T. cruzi* infection in humans have historically been associated with the presence of *Triatoma infestans* (Klug, 1834), which is native to Bolivia and was introduced and spread in Brazil via migratory flows^10^. Evidence indicates that *T. infestans* tends to outcompete native species and become predominant within two to three years, as demonstrated in Minas Gerais by Martins, Versiani, and Tupinamba in the 1940^11,12^.

*Panstrongylus megistus* holds both epidemiological and historical significance, as it was in this species that Carlos Chagas first discovered and described the parasite *T. cruzi*. At that time, *P. megistus* was the only domiciliated triatomine species in the municipality of Lassance, in Minas Gerais State. Subsequently, *T. infestans* reached this region and became the dominant triatomine species^13^, a scenario that was replicated in the municipality of Bambui, also in Minas Gerais, in the late 1930s^12^.

Following the demonstration of the efficacy of benzene hexachloride for triatomine control, the so-called Chagas Disease Prophylaxis Program conducted vector control activities in Brazil until 1968. However, these efforts were irregular, inconsistent, and lacked continuity across the geographical area requiring intervention^14^. Acknowledging the fragmented nature of the available information derived from poorly integrated and haphazard initiatives, the Brazilian Ministry of Health decided to establish a baseline to guide large-scale control measures. As a result, several major national surveys were undertaken, including an entomological survey conducted between 1975 to 1983^15^ and a seroprevalence survey conducted between 1975 and 1980^16^.

This study cross-referenced data from national surveys conducted between 1975 and 1983 to investigate the roles of the most epidemiologically significant triatomine species in Minas Gerais, *P. megistus* and *T. infestans*, as vectors of *T. cruzi* during a historical period of intense transmission in the region. The analysis was based on entomological surveys and seroprevalence data from the Divinopolis Regional Health Superintendence (SRSD) in Minas Gerais State, Brazil.

## MATERIALS AND METHODS

### Study area

**Figure 1:**
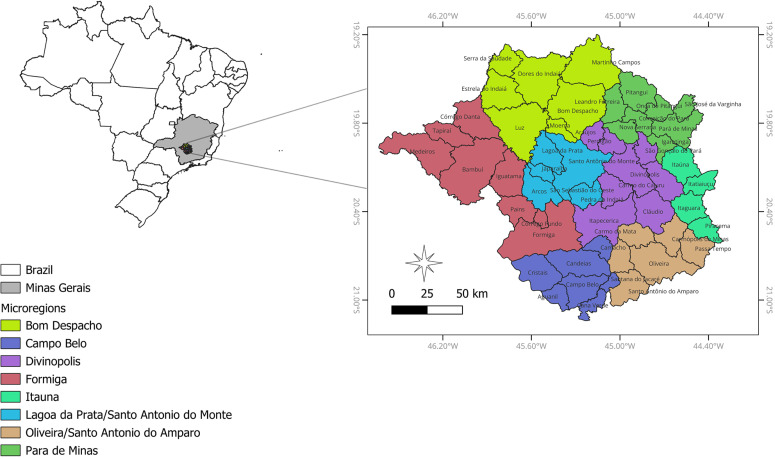
The eight health microregions and their respective municipalities within the Divinopolis Regional Health Superintendence study area, showing the 52 municipalities that existed prior to 1995 and during the study period (1975 and 1983), in the mid-west region of the Minas Gerais State, Brazil.

The study area is located in the midwest region of the state of Minas Gerais, Brazil, and currently comprises 53 municipalities under the jurisdiction of the Divinopolis Regional Health Superintendence (Figure 1). However, the municipality of Córrego Fundo was established in 1995, following its separation from the municipality of Formiga. Therefore, the analyses in this study are based on the 52 municipalities that existed prior to the creation of Córrego Fundo. These municipalities are organized into eight health "microregions": Bom Despacho, Campo Belo, Divinopolis, Formiga, Itauna, Lagoa da Prata, Oliveira, and Para de Minas, Figure 1.

### Data source

Data from the published entomological survey reporting the geographic distribution of all the triatomine species captured, by municipality, across the national territory between 1975 to 1983 were used to assess the occurrence of *P. megistus* and *T. infestans* ^15^. From these data, a specific subset was created for the municipalities comprising the SRSD, focusing exclusively on the occurrence of *P. megistus* and *T. infestans*. Similarly, prevalence data for Chagas disease in the SRSD municipalities between 1975 to 1980 were extracted from the published serological survey for that period^16^. The seroprevalence of human *T. cruzi* infection in each municipality was categorized into three groups: low (<10%), intermediate (>10% to <16%), and high (>16%).

### Data analysis

The geographic coordinates of the 52 municipalities were obtained from the IBGE^17^. Thematic maps were generated to visualize the presence/absence of the two triatomine species in each municipality within the study area. Additionally, gradient maps were created to illustrate the seroprevalence of human *T. cruzi* infection across the SRSD region. All maps were constructed using QGIS software (version 3.30).

## RESULTS

**Figure 2:**
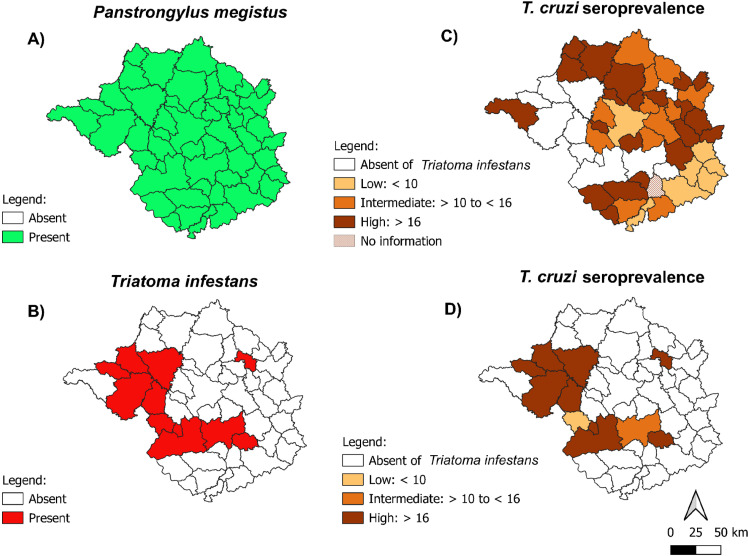
Comparison of the geographic distributions of the occurrence of *Triatoma infestans* and *Panstrongylus megistus*, and the seroprevalence of human *Trypanosoma cruzi* infection, in the municipalities of the Divinopolis Regional Health Superintendence study area in the mid-west region of Minas Gerais State, Brazil, between 1975 and 1983: (A) The absence and presence of *P. megistus*; (B) The absence and presence of *T. infestans*; (C) The seroprevalence of human *T. cruzi* infection (%), in the municipalities where *T. infestans* was absent, and only *P. megistus* was found; (D) The seroprevalence of human *T. cruzi* infection (%), in the municipalities where both *T. infestans* and *P. megistus* were found.

Data from Silveira *et al*.^15^ showed that *P. megistus* had a broad geographical distribution, being present in all studied municipalities (100%, 52/52) (Figure 2A). In contrast, *T. infestans* was detected, and therefore co-existed with *P. megistus*, in only 19.2% (10/52) of the municipalities (Figure 2B).

In the 41 municipalities where only *P. megistus* was detected and for which relevant epidemiological data were available, the mean seroprevalence of human *T. cruzi* infection was 17.8%, ranging from 1.0% to 41.9% (Figure 2C). In the 10 municipalities where *T. infestans* occurred, the mean seroprevalence was higher, at 25.8%, with a range of 9.8% to 40.8% (Figure 2D). Among the 41 municipalities with only *P. megistus* and available data, more than 50% were classified as having high seroprevalence. In contrast, 80% of the municipalities with *T. infestans* infestation were classified as having high seroprevalence of human *T. cruzi* infection (Table 1).

**Table 1:** Comparison of the occurrence of *Triatoma infestans* and *Pantrongylus megistus* according to the level of the seroprevalence of human *Trypanosoma cruzi* infection in the municipalities of the Divinopolis Regional Health Superintendence (SRSD) in the midwestern region of Minas Gerais State, Brazil, between 1975 and 1983.

*Trypanosoma cruzi* seroprevalence (%)	% (number) municipalities species present	
	*Pantrongylus megistus only*	*Triatoma infestans* + *Pantrongylus megistus*
Low: < 10	19.5% (8/41)	10.0% (1/10)
Intermediate: > 10 to < 16	29.3% (12/41)	10.0% (1/10)
High: > 16	51.2% (21/41)	80.0% (8/10)

## DISCUSSION

The western region of Minas Gerais State has historically been recognized as an endemic area for Chagas disease, which is significant in the history of the region. Several triatomine species have been consistently reported in the area since the 1970s, including *Panstrongylus diasi* Pinto & Lent, 1946; *Panstrongylus geniculatus* (Latreille, 1811); *Rhodnius neglectus* Lent, 1954; *Triatoma sordida* (Stål, 1859); and *Triatoma pseudomaculata* Corrêa & Espínola, 1964, with *P. megistus* remaining particularly significant due to its persistence^18,19^.

*Panstrongylus megistus* was the predominant triatomine species in Minas Gerais until the end of the 1930s, accounting for 79.8% of the reported distribution, including within the region now corresponding to the SRSD. During this period and shortly thereafter, *T. infestans* spread rapidly and extensively across Brazil, eventually surpassing *P. megistus* and becoming the dominant species by the early 1940s^12^, when it became the main vector in the state. In the 1970s, the natural infection rate was 8.7% for *T. infestans* and 3.4% for *P. megistus* ^20^.

Previous studies have reported the coexistence of both species in Minas Gerais State^15,21^. Although the geographical distribution of *T. infestans* was more restricted than that of *P. megistus*, the intradomestic colonies of *T. infestans* generally had more individuals^21–23^, highlighting the competitive advantage of this exotic species over native triatomines within domestic environments^24^. The spread of *T. infestans* across Brazil displaced native species, leading it to become the predominant vector in its areas of occurrence and the greatest challenge for controlling Chagas disease transmission^13^.

*Panstrongylus megistus* was previously reported in 415 municipalities in Minas Gerais^15^ and was widely distributed across all municipalities within the SRSD, as also confirmed in this retrospective analysis. A comparison between the data from Silveira *et al.* ^15^ and the one published by Villela *et al*.^18^ for the same study area revealed that *P. megistus* remained the most widely distributed species between 2003 to 2007 and was also the most frequently captured triatomine during that period.

The biological behavior of *P. megistus* supports its broad dispersion, as this species shows strong capacity for adaptation to both peridomestic and intradomestic environments. Typically, it has a single peak of adults during the rainy season, which is consistent with the occurrence of a single annual cycle^23,25,26^. Adults of *P. megistus* disperse from October to February, with frequent reports of insects invading homes and establishing new foci, coinciding with a time when residents are at increased risk of exposure to *T. cruzi*. In contrast, in areas subjected to sustained vector control over the past 30 years, where triatomine colonies are generally maintained at low densities, dispersal events from domestic units tend to be infrequent or rare^27^.

Among the municipalities where only *P. megistus* was present, 51.2% recorded a high seroprevalence of human *T. cruzi* infection (>16%), demonstrating the vectorial potential of this species in the absence of *T. infestans*. In general, *P*. *megistus* shows high rates of natural infection with *T. cruz*,^13,28,29^. Furthermore, this species can blood-feed on a broad range of vertebrate hosts, including humans (22.5%), which facilitates its adaptation to novel anthropogenic environments^19^. In previous studies, as is typical for triatomines that colonize peridomestic environments, birds—particularly chickens—were identified as the primary food source in this region. However, the proportion of insects feeding on human blood further underscores the anthropophilic behavior of *P. megistus* ^19,28^.

## CONCLUSION

The seroprevalence of human *T. cruzi* infection exceeded 16% in 80% of the municipalities where the *T. infestans* was present, highlighting its significant vectorial potential, which likely contributed to Minas Gerais State having the highest number of people infected by *T. cruzi* at the time^15^. *T. infestans* was the primary target of the Chagas Disease Control Program (PCDCH) until vector transmission by this species was eliminated in Brazil in 2006^30^. Following its elimination, *P. megistus* re-emerged as the most important vector of *T. cruzi* in Minas Gerais State^19,31^, and currently represents the major challenge for triatomine control in municipalities within the SRSD.

DATA AVAILABILITY

The complete anonymized dataset supporting the findings of this study is included within the article itself.
